# Emotional Reactions of Mothers Facing Premature Births: Study of 100 Mother-Infant Dyads 32 Gestational Weeks

**DOI:** 10.1371/journal.pone.0104093

**Published:** 2014-08-25

**Authors:** Julien Eutrope, Aurore Thierry, Franziska Lempp, Laurence Aupetit, Stéphanie Saad, Catherine Dodane, Nathalie Bednarek, Laurence De Mare, Daniel Sibertin-Blanc, Sylvie Nezelof, Anne-Catherine Rolland

**Affiliations:** 1 CHU Reims, Hôpital Robert Debré, Structure interne de Psychothérapie de l'Enfant et de l'Adolescent, Reims, F-51092, France; 2 CHU Reims, Hôptal Robert Debré, Unité d'aide méthodologique, REIMS, F-51092, France; 3 Centre d'Action Médico-Sociale Précoce, Reims, F-51100, France; 4 CHU Nancy, Centre psychothérapique de Nancy, Service de pédopsychiatrie, Laxou, F-54521, France; 5 CHU de Besançon, Hôpital Saint Jacques, Service de psychiatrie de l'enfant et de l'adolescent, Besancon, F-25030, France; 6 CHU de Reims, Hôpital Maison Blanche, Structure Interne de Pédiatrie B, Reims, F-51092, France; Chiba University Center for Forensic Mental Health, Japan

## Abstract

**Objectives:**

This current study has been conducted to clarify the relationship between the mother's post-traumatic reaction triggered by premature birth and the mother-infant interactions. In this article, the precocious maternal feelings are described.

**Methods:**

A multicenter prospective study was performed in three French hospitals. 100 dyads with 100 very premature infants and their mothers were recruited. Mothers completed, at two different times self-questionnaires of depression/anxiety, trauma and social support. The quality of interactions in the dyads was evaluated.

**Results:**

Thirty-nine percent of the mothers obtained a score at HADS suggesting a high risk of depression at the first visit and approximately one-third at visit two. Seventy-five percent of the mothers were at risk of suffering from an anxiety disorder at visit one and half remained so at visit two. A “depressed” score at visits one and two correlated with a hospitalization for a threatened premature labor. We noted a high risk of trauma for 35% of the mothers and high interactional synchrony was observed for approximately two-thirds of the dyads. The mothers' psychological reactions such as depression and anxiety or postnatal depression correlate strongly with the presence of an initial trauma. At visit one and visit two, a high score of satisfaction concerning social support correlates negatively with presence of a trauma. A maternal risk of trauma is more frequent with a C-section delivery.

**Conclusions:**

Mothers' psychological reactions such as depression and anxiety correlate greatly with the presence of an initial trauma. The maternal traumatic reaction linked to premature birth does not correlate with the term at birth, but rather with the weight of the baby. Social support perceived by the mother is correlated with the absence of maternal trauma before returning home, and also seems to inhibit from depressive symptoms from the time of the infant's premature birth.

## Introduction

In January 2008, in an article titled, “Preterm: What Can be Done?”[1], The Lancet announced a series of three articles series by Robert Goldenberg, to focus on a rising public health issue by studying causes as well as interventions to reduce infant morbidity and mortality, which according to the author was being neglected. According to this editorial, premature births account for 12–13% of births in the United States, and 5–9% in a number of other developed countries. Remarkably, the numbers of very premature births has increased over the last decade. An epidemiological birth study from 2005 [2] states that the United States has a premature birth rate (<37 Gestational Weeks) of 12.7% compared to 10.6% in 1990 and only 9.5% in 1981. Another objective study [3] also shows a 20% rise in premature births between 1990 and 2005. In Europe, the rate increased by 22% [4], concerning first-time mothers in particular, a trend also found in Australia [5]. The rise in medically assisted procreation partly explains this increase in which mothers pregnant with multiples are often induced or have ovary stimulation [6]. The risk of a very preterm birth is considerably higher in the case of multiples: in 1995 7% of twins and 25% of triplets were born before 33 weeks. Furthermore, cases of prematurity are higher in single pregnancies done with in vitro fertilization.

In this article we want to discuss very preterm births or births occurring before 32 Gestational Weeks (GW). An infant, who is born very preterm, worries neonatologists as to his/her viability and his/her pathologies for the short-term outlook. Premature births are considered to be responsible for 75% of perinatal mortality, and more than 75% of the long-term perinatal morbidity [7].

Our intervention in the maternity and neonatal wards sensitized us to the immediate parental reaction of the premature birth. Among these reactions, most often the parents are what they call “stressed”, which is the origin of trauma. The unexpected confrontation with a baby is far from what the parents had anticipated; the shock experienced during a rapid chain of events taking place, an experience of emptiness when the baby is placed in the intensive care unit, the feeling of powerlessness when facing the real risk of the infant's death, the invasive treatments, etc. The parents worry about the viability and future of their premature infant. Their parental reactions and more specifically those of the mother facing the premature birth could have repercussions on the development of the infant, due to complex and atypical interactions.

These observations have driven us to elaborate on a hypothesis that distinguishes itself separate to the lesion model commonly applied to premature infants, and shifts the psychopathological approach to the post-traumatic reaction of the mother following the premature birth; it repositions the question of behavioral disorders of the child in the mother-infant interactions. In a second phase, we think that a premature birth can bring about post-traumatic symptoms as described in the post-traumatic stress state, and most notably in the mother. Therefore, this reaction could have effects on the mother-infant interactions.

We have conducted this current study to clarify the relationship between the mother's post-traumatic reaction triggered by the premature birth and the mother-infant interactions. In this article, our research aims to describe precocious maternal feelings when the mother has to face the premature birth of her infant.

## Methods

### 1. Design

This present study is part of the larger research program that was a multicenter prospective study performed in 3 French hospitals (Reims, Nancy and Besançon) between January 2008 and January 2011. The follow-up period was 18 months for each participant. The entire research program covers 5 visits: the first visit, in the maternity service following the birth of the child, and the second visit, in the neonatology service, right before the discharge. The three following visits with the same design took place at 6, 12 and 18 months, within the framework of the systematic tracking of premature babies within the early medico-social actions' center (CAMSP). The study described in this article analyses the results of the first two visits of the research program.

### 2. Sample and procedure

All the preterm infants who were admitted to one of the NICU of the 3 investigation centres (Reims, Besancon and Nancy) from January 2008 to January 2010 and who matched our principal inclusion criteria (<32 gestational weeks) were considered for inclusion in the study. Exclusion criteria were for the mothers with an evident psychiatric illness, drug or alcohol abuse, aged <18 years, and language barriers; for the newborn: unfavourable vital prognosis evaluated with the Perinatal Risk Inventory [8] (a PRI score ≥10 identifies infants at risk of significant developmental disabilities and constitutes as an infant exclusion criteria), malformation and/or genetic anomaly diagnosed before the inclusion.

The 3 maternities and NICU are levels 3B (a NICU that provides mechanical ventilation without restrictions but does not provide major surgery) or 3C (a NICU that provides major neonatal surgery but neither open-heart surgery nor extracorporeal membrane oxygenation). These definitions are based on the American Academy of Paediatrics report on NICU levels of care [9]. Regarding the location of the family's city, 30% of the infants considered for inclusion were excluded. Undoubtedly the area of the family had to be close to the investigation centre to allow the routine follow-up proposed to preterm infants linked to our study. For the mother exclusion criteria concerning a psychiatric illness, we based it on a standard clinical interview performed by a psychiatrist investigator responsible for the inclusions. For the infant exclusion criteria, pediatricians connected to NICU recorded the PRI (Perinatal Risk inventory) and decided whether to allow the inclusion or not. To consider infant and mothers exclusion criteria, it excluded 20% of the dyads divided as: for the mothers: evident psychiatric illness (n = 4), drug or alcoholic abuse (n = 2), age <18 years (n = 2) and language barriers (n = 7); for the newborn: PRI ≥10 (n = 60), malformation and/or genetic anomaly diagnosed before the inclusion (n = 5). Following these restrictions of location and exclusion criteria, this study was explained and proposed to 50% of the population relating to our principal inclusion criteria (birth <32GW). Sixty-five percent of the mothers accepted to participate in this study (130 mothers). There were 26 cases of multiple births (22 twins, and 4 triples); only one child (randomly selected) was kept for the study; 30 children were then removed. Limited by our financial funding, we decided to stop the inclusions when reaching a population of 100 dyads, a number considered high enough by the statisticians. The second visit was completed by 93 dyads, 2 infants died, 2 infants were transferred to another hospital ward because their family moved away and 3 mothers dropped out.

### 3. Ethics

Given the observational nature of the study, which is not governed by French biomedical law, it could not be approved by the Committee for the Protection of Persons. Given the guidelines of publishers focused on the ethics of the investigations published, CHU de Reims has established an IRB since 2007. This study has been approved globally for all the centers (Reims, Nancy and Besançon) and all the participating patients. It received the approval of the local Institutional Review Board: IRB of Reims University Hospital. The study was conducted in compliance with good clinical practice, as indicated in the Declaration of Helsinki. Written consent was obtained from all the patients after providing specific information on the study. After a premature birth below 32 gestational weeks, NICU pediatricians contacted our research team and one of the practicians/author in charge of the clinical part of the research, met the mother and her baby to introduce the research program. Therefore, some of the authors have been in contact with participants as expected in a clinical research. Patients were free to refuse to take part in the study or to withdraw from it at any stage on simple request, without any alteration to the care provided to them. Data collection was made anonymous.

### 4. Data collection

#### 4.1 Sociodemographic and clinical data

Sociodemographic variables recorded during the study were age, marital status, level of education and profession.

For the mothers, clinical data were: number of childbirths, number of pregnancies, medical history of the pregnancy (threatened premature labor, hospitalization), obstetric history (miscarriage, medical interruption of pregnancy, termination of pregnancy, in vitro fertilization, threatened premature labor, prematurity, hospitalization), delivery conditions (C-section or vaginal), multiple pregnancy (number of babies), anesthesia type (none, epidural, total), personal histories (medical, surgical, psychiatric, family).

For the infants, clinical data were: date of birth, term of birth, weight, size, cranial perimeter, Apgar score at five and ten minutes, necessity or not of a neonatal resuscitation, method of feeding (breast-, bottle-, mixed-feeding).

For the dyads, clinical data was the presence or absence of specific care (psychologist, psychomotor therapist).

#### 4.2 Measures

The design of this study's two visits is described in table 1.

**Table 1:** Design of the two first visits of the study.

	Place	Date	PPQ	HADS	EPDS	SSQ	PRI	DMC
Visit 1	Maternity	Day1 to day 15		X		X	X	
Visit 2	Neonatalogy	15 days before output	X	X	X	X	X	X

For the mothers, the evaluation of the mother's trauma was done by using *mPPQ (modified PTSD-Perinatal Posttraumatic Stress Disorder-Questionnaire*) [10], [11]. This self-report questionnaire consists of 14 items, specially adapted to the parents of perinatal high-risk children, in order to evaluate the presence of traumatic elements concerning the birth. For this measurement, we decided to use a cut-off score of ≥19 [10], [12], which identifies a high risk of trauma in the maternal population needing specific care (referral for therapy) when the PPQ has reached 19 or more.

In order to compensate for the biases identified, we also evaluated the maternal co morbidity using:

The *HADS (Hospital Anxiety and Depression Scale)* [13], [14], which is a self-report questionnaire frequently used in international literature, and divided into 2 sub-scales, which are anxiety and depression HADS scales. This scale, which is easy to use, makes it possible to evaluate anxious and depressive symptoms in people who have somatic diseases. It allows episodes of recent anxiety and depression to be evaluated and to attribute to an overall score for each one. The result is given as a score. We have used a cut-off score of 8 or above, which is suggestive of clinically significant anxious (HADS anxiety sub-scale) or depressive (HADS depression sub-scale) symptomatology [15].

The *EPDS (Edinburgh Post-natal Depression Scale)* [16] consists of a self-report questionnaire that tracks postnatal depression and includes 10 items, which we propose to the mother from the fourth week of postpartum. A score of 12 or above is suggestive of clinically significant depressive symptomatology. The EPDS cutoff has been found to identify major depression in women, with a sensitivity of 86%, a specificity of 78%, and a positive predicative value of 73% [17].

*The Social Support Questionnaire (SSQ) of Sarason* [18] is a self-report questionnaire which includes 2 scales: availability (the number of people of whom the subject can rely on), and satisfaction (the level of fulfillment concerning this support).

For the infants, we identified some who were at risk of significant developmental abnormalities by using the *Perinatal Risk Inventory (PRI)* [8]. This scale uses 18 items to describe the weight of the perinatal problems and the severity of the perinatal risk, based on perinatal factors such as the Apgar score, the gestational age, the weight or the cranial perimeter. The score can be from o to 51. An inventory score ≥10 identifies infants at risk of significant developmental disabilities. The performances of the Perinatal Risk Inventory are: sensitivity 76%, specificity 79%, positive predictive value 47,7%, and negative predictive value 92,9%.

In the dyad, Censullo's DMC (Dyadic Mutuality Code) instrument [19] can be used for the first 6 months of the baby's life. After a 5-minute observation of the dyad interacting, the observer codes 6 items (mutual attention, positive affect, reciprocity, maternal pauses, clearness of the signals of the baby, and receptivity/maternal sensitivity). A score of 1 or 2 is given for each item and a total score is rated as synchronous or low synchronous. The total score ranges from 6 to 12. A score of 6 to 9 is ranked as low synchronous; and 10 to 12, as synchronous. The summary ratings use combinations of all 6 items as an operational definition of the levels of synchrony, because included in the 6 items are the relevant components of synchronous interaction established in specialized literature, and collectively they represent the concept of synchrony. The validation of the DMC has been constructed by Censullo on relevant findings of interrater reliability, items analysis, concurrent to 38 videotapes of mother-infant face-to-face interaction, compared to cross-spectral analyses of the Monadic Phase Scale [20].

### 5. Statistical analysis

The quantitative variables are the average, the standard deviation, and the minimal and maximal values. The qualitative variables are the number of observations and the associated frequency. The search for a link between the quantitative and qualitative variables was accomplished by using the Mann-Whitney/Wilcoxon or Kruskal-Wallis test. We did Spearman's correlation analysis between two quantitative variables. For all statistical tests, a p value<0.05 was considered statistically significant. Analyses were performed using the SAS software (version 9.0; SAS Institute Inc., Cary, N.C., USA).

## Results

### 1. Patient characteristics

We included 100 dyads (100 babies and their mothers). The average age of the mothers is just under 30 years old (29.8±6 [17–45], and 92% of them lived with a partner at the time of birth. The majority have a higher education (79.29%) and work (69%). Most of the mothers (88.8%) have been hospitalized for a risk of premature labor. The majority of them (58.2%) have obstetrical antecedents (miscarriage, medical termination of pregnancy, IVF, premature birth). It is generally a first (48%) or a second pregnancy (32%), with a small majority of births by C-section (54%) and a rather homogeneous distribution of the method of feeding (40.82% breast-, 33.67% bottle-, 25.51% mixed-feeding). The average term is from 29 weeks of amenorrhea and 6 days (29.84±6 [17–45], with a very small majority of boys and an average birth weight of 1320g.

### 2. Mother evaluation

Table 2 represents the scores of evaluation scales at visit 1 and visit 2.

**Table 2:** Mean scores of evaluation scales at visit 1 and visit 2.

	N^a^	m (sd)^b^
*Visit 1*	*100*	
PRI^1^	95	4.7 (2.5)
HADS^2^ Depression	98	6.8 (4.3)
HADS^2^ Anxiety	98	10.2 (4.4)
HADS^2^ Global	98	17.0 (7.9)
SSQ^3^ Disponibility	98	21.6 (12.3)
SSQ^3^ Satisfaction	98	31.5 (4.8)
*Visit 2*	*93*	
PRI^1^	72	6.8 (7.1)
HADS^2^ Depression	89	5.4(3.2)
HADS^2^ Anxiety	89	8.5 (3.7)
HADS^2^ Global	89	13.9 (6.2)
SSQ^3^ Disponibility	87	22.0 (12.6)
SSQ^3^ Satisfaction	88	31.9 (4.3)
mPPQ^4^	88	16.4 (9.9)
EPDS^5^	89	9.2 (5.1)
DMC^6^	90	10.2 (1.2)

a N: Number of dyads.

b m(sd): mean (standard deviation).

1 PRI: Perinatal Risk Inventory.

2 HADS: Hospital Anxiety and Depression Scale.

3 SSQ: Social Support Questionnaire of Sarason.

4 mPPQ: modified Perinatal PTSD (Post Traumatic Stress Disorder) Questionnaire.

5 EPDS: Edimburgh Postnatal Depression Scale.

6 DMC: Dyadic Mutuality Code.

Our first results show that 39% of the mothers obtained a score at HADS suggesting a high risk of depression at the first visit and approximately one-third at the second visit (HADS depression sub-score: 26%, EPDS: 34%). HADS anxiety sub-score reveals that 75% of the mothers were at risk of suffering from an anxiety disorder at visit one and half (50%) were still at risk at visit two [21]. A high score at visits one and two correlated with a hospitalization for a risk of premature labor (p_V1_ = 0.0392; p_V2_ = 0.0282) and did not correlate with the babies' birth term (p = 0.64) or birth weight (p = 0.55) at visit two.

We noted a high risk of trauma in 35% of the mothers and a high interactional synchrony score was observed for approximately two-thirds (66%) of the dyads.

Access to services to a psychologist or a psychomotor therapist was offered to 30.1% of the mothers and their babies.

In Table 3, the data concerning the mothers' and babies' clinical characteristics correlated significantly with the mother's trauma.

**Table 3:** Mothers' and babies' clinical characteristics and the high risk of maternal trauma.

	High risk of initial trauma^3^r (p)
State of health of the child (PRI to V2)	0.25 (0.04)
Evolution of the state of health of the child (PRI V2 - PRI V1)	0.25 (0.04)
Birth Weight	−0.23 (0.03)
HADS^1^ depression score V1	0.54 (<.0001)
HADS^1^ depression score V2	0.48 (<.0001)
HADS^1^ anxiety score V1	0.54 (<.0001)
HADS^1^ anxiety score V2	0.52 (<.0001)
HADS^1^ global score V1	0.60 (<.0001)
HADS^1^ global score V2	0.56 (<.0001)
Satisfaction of perceived social support (SSQ Satisfaction V1)	−0.23 (0.03)
Satisfaction of perceived social support (SSQ Satisfaction V2)	−0.22 (0.04)
EPDS^2^ (postnatal depression V2)	0.50 (<.0001)

1 HADS = Hospital Anxiety and Depression Scale.

2 EPDS = Edimburgh Postnatal Depression Scale.

3 Score at modified Perinatal PTSD (Post Traumati Stress Disorder) Questionnaire (mPPQ) ≥19 suggesting a maternal trauma.

The mothers' psychological reactions at visit one and two such as a high risk of depression and/or anxiety disorder or postnatal depression at visit two correlated strongly with the high probability of initial trauma (p<0.0001).

At visit one (p = 0.03) and visit two (p = 0.04) a high score of satisfaction concerning social support correlates negatively with a high score on the PPQ scale (which indicates a mother's trauma). A high score obtained on the PPQ is significantly (p = 0.0092) more frequent with a C-section delivery (vs. vaginal delivery) and the majority of the mothers with a high risk of trauma (PPQ's high score) belong to the dyads having specific psychological care with psychologists or psychomotor therapists (p = 0.0026).

### 3. Baby evaluation

The babies' health characteristics correlate with the presence of an initial trauma: state of health of the newborn (PRI score to V2) (p = 0.04), his or her's evolution of state of health (p = 0.04) and his or her birth weight (p = 0.03).

### 4. Dyad interaction

We looked for correlations between the quality of interactions in the dyads (evaluated with the DMC on Visit 1 and 2) and the characteristics collected on Visit 1 as: pregnancy conditions (medically assisted procreation or not), delivery conditions (C-section or vaginal), babies' clinical characteristics (birth weight, intrauterine growth restriction, term at birth), and the infant's measures (PRI score), and also the mothers' measures (HADS, EPDS and PPQ scores) for evaluating states of mind of the mothers; but we did not find any significant correlation.

## Discussion

### Prerequisite

As clinicians, it's essential to underline the complexity of the subject. Prematurity, depending on the term at birth, takes many different forms. Many innate and acquired factors, outside of the gestation period itself, intervene in clinical prematurity. It would be simplistic and inexact to consider clinical prematurity as a well-defined entity. Also, for facilitating the reading, in this discussion, we will use the terminology of depressed or anxious for mothers with high scores on specific self questionnaires (HADS and EPDS), allowing them enough substantial to assess anxious and depressive symptomatology, especially by using the cutoff mentioned in the method paragraph.

The general characteristics (sociodemographic and clinical data, main psychological reactions) of our population were comparable with other studies also addressing the maternal reactions after a premature birth [22]–[24].

We didn't find any significant correlations between maternal distress and dysfunctional interactions, whereas our hypothesis was that a maternal traumatic reaction would be correlated with difficulties in parent-child interactions, especially as the general sense of this hypothesis has been partially confirmed by some studies [25]–[27]. By the second visit we observed the mother-infant interactions, but no significant correlation was found between low-quality interactions nor with certain maternal emotional reactions such as traumatism, or with any of the factors related to the child (term, weight, health). It seems that the assessment tool (DMC) is not sensitive enough. We could also question the timing of the observation at visit two, which is just before the infant returns home so the mother is very enthusiastic and in a hurry to return home. It probably would have been more efficient to observe interactions at home.

Nonetheless, the findings of this study about precocious, maternal and emotional reactions are interesting and consistent with previous studies which suggest that the premature birth of an infant has repercussions on maternal emotional responses [28]. In many studies, elevated scores affirming postnatal depression were found to be significantly more often associated with the mothers of infants born prematurely [22], [29]. In this study, during the 15 days after birth, 39% of the mothers attained scores on the HADS that suggested a high risk of depressive symptoms and about one third of the mothers remained at risk of depression during the few days before the discharge from the NICU. This evolution of depression scores approves with an American longitudinal study [30] in which more than half of the population of mothers with premature babies were considered depressed (60%) according to the study criteria, with a progressive improvement of the symptomatology reaching stability at the infant's sixth month. The correlation between positive scores of depression's risk and threatened premature labor confirm the results of previous studies [31], [32] which find psychological origins in more than 50% of those at risk of premature labor.

A large majority of mothers (75%) present a post-natal symptomatology of anxiousness in the early post-natal period following the premature birth of their child and before their child goes home. The results are in concordance with a New Zealand study [33] that finds that anxiety dominated in the group where parents were confronted with the hospitalization of their infant versus the second group where the infants were never placed in the NICU. However, in the first group, a significant difference was found depending on the reason for the infant's hospitalization: premature births lead to more depressive symptomatology in NICU admissions than infants that were born at term. The premature birth itself plays its own role, independent of only stress linked to the infant's state of health.

Traumatism, measured by the PPQ scale after the birth and before the return home, is objective among 35% of the mothers. Our results confirm those of previous studies [23], [24]. For all of the parents, the premature birth of their infants is an intense experience, shattering their psychological reserves. A one year French prospective study [34] shows that the feeling about this experience changed little during the infant's first year of life: memories remain vivid and raise strong emotions. Certain parents retell the birth story using exactly the same words with the same effects one year later. A birth like this could therefore be considered as a traumatic event. In an article co-written by Léon Kreisler and Michel Soulé [35], the mothers' emotional response and the initial circumstances surrounding the premature infant is studied by evaluating the pregnancy, delivery, separation, and subsequent evolution. This is a psychological storm that bears on the mother. Once objectified, the presence and extent of this maternal trauma reaction following the premature birth of her child makes it important to attempt to correlate certain factors. According to our results, the method of delivery, and more specifically when a cesarean occurs, correlates significantly with signs of a traumatic reaction, objectified and quantified during the second visit of our research protocol. The same was also found regardless of the term at birth [36], [37].A Norwegian study [38] shows that the mothers having undergone a caesarean section are 12% less likely to have a second baby than other mothers. We can reasonably assume that the psychological trauma of a cesarean section therefore remains an obstacle into having another pregnancy. A premature birth and delivery requiring a cesarean section would be two factors predisposing parturient women to the development of a traumatic reaction.

According to our results and the results of another recent study [23], the maternal traumatic reaction linked to the premature birth does not correlate to the term at birth, but rather to the weight of the baby. This point is also witnessed in medical consultations, even a long time after the premature birth, because the parents, or sometimes even the child, report the weight to indicate the severity of the birth and their worries, and not the term. Parents remain sensitive to the news of “low weight” at birth (500, 600 grams), and also by the image of this weight, which seems to actively participate in the emergence of an anxious and traumatic symptomatology in the mother. We have also looked for a correlation with the presence of an intrauterine growth restriction (IUGR) by using applicable scales. We know that in terms of developmental evolution of a premature infant, the existence of intrauterine growth restriction increases the risk of mortality, morbidity, and sequelae, because of chronic anoxia, cerebral malnutrition, and even the causes that led to the IUGR. But we did not find any correlation between the negative maternal emotional responses and the existence or lack of IUGR. Only the infant's weight at birth, a factor perceived visually by the mother, is a factor of the emergence of the mother's negative emotions.

Our results show a correlation between the somatic state of health of the baby quantified by the Perinatal Risk Inventory (PRI), and the maternal trauma (measured by the PPQ). During the premature infant's hospitalization in the NICU, the parents are worried and are constantly listening to the information being relayed to them several times a day by the professionals in regards to the state of their infant's health. Daniel Sibertin-Blanc [39] describes certain premature infant's parents as vigilant guardians of their infant's bodies. Catherine Druon [40] alludes to the possible risk of passing from a “maternal preoccupation” to a “primarily medical preoccupation”. Submerged in an atypical medical universe, mothers learn how to become good technicians, leaving aside normal maternal preoccupations.

Social support perceived by the mother, both in the number of people recognized to support the mother and the quality of this support is essential and is significantly correlated with the absence of maternal trauma before returning home, and also seems to protect from depressive symptoms from the time of the infant's premature birth.

One of the most interesting results on the clinical level of our research involves the support given to mothers in neonatal units. The identified mothers as having psychological distress in the aftermath of the premature birth through the study's questionnaires (PPQ, HADS) are those who have benefited from support. They benefit from care by a psychologist or psychomotor therapist during the hospitalization of their infant in the NICU, without the results of their self-questionnaires being known by professionals.

## Limitations

By not using structural interviews for the mothers' assessments at the time of the inclusion to respond categorically to the mother exclusion criteria, and also to diagnostic depression symptoms in the two visits, is a main limitation of our study. However, structured assessments might have been too intense, even too emotionally difficult for the mothers having just experienced the premature birth of their infant, and have to continue to live with this supposed traumatic event. About the representation of our population, the location restrictions (explained in the “Sample and Procedure” paragraph), could appear as a limitation to our study, because our population most probably live in big cities or nearby. Concerning our data collection, we didn't have any data concerning mother anxiety and or depressive symptoms before the infant's birth, so this remains a limitation for us to discuss our results. Also it would have been pertinent to investigate the mother's smoking habits, so as to correlate to the infant's characteristics. At least, as for any study, a control group would have permitted the results to be more consistent.

## Conclusion and Clinical Implications

The mother is the central figure of anxiety related to premature birth. She experiences and expresses her feelings about it (not necessarily), and it is up to the clinical staff to hear what she is feeling. In our study, we observed that professionals have been sensitive to the expression of maternal suffering, and have proposed specific support. This kind of intervention is now well described in literature (38), so we believe that being able to express suffering is a good prognosis in the evolution of trauma for the mother.
